# Endoplasmic Reticulum Stress in Osteoarthritis: A Novel Perspective on the Pathogenesis and Treatment

**DOI:** 10.14336/AD.2022.0725

**Published:** 2023-04-01

**Authors:** Zeqin Wen, Qi Sun, Yunhan Shan, Wenqing Xie, Yilan Ding, Weiyang Wang, Ruixi Ye, Wenfeng Xiao, Yusheng Li

**Affiliations:** ^1^Department of Orthopedics, Xiangya Hospital, Central South University, Changsha, China.; ^2^Xiangya School of Medicine, Central South University, Changsha, China.; ^3^National Clinical Research Center for Geriatric Disorders, Xiangya Hospital, Central South University, Changsha, China.; ^4^Department of Orthopaedics, Shanghai Tenth People's Hospital, Tongji University School of Medicine, Shanghai, China.

**Keywords:** osteoarthritis, endoplasmic reticulum stress, cartilage degradation, pathogenesis, treatment

## Abstract

Osteoarthritis (OA), the most common degenerative joint disease, causes an enormous socioeconomic burden due to its disabling properties and high prevalence. Increasing evidence suggests that OA is a whole-joint disease involving cartilage degradation, synovitis, meniscal lesions, and subchondral bone remodeling. Endoplasmic reticulum (ER) stress is the accumulation of misfolded/unfolded proteins in the ER. Recent studies have found that ER stress is involved in the OA pathological changes by influencing the physiological function and survival of chondrocytes, fibroblast-like synoviocytes, synovial macrophages, meniscus cells, osteoblasts, osteoclasts, osteocytes, and bone marrow mesenchymal stem cells. Therefore, ER stress is an attractive and promising target for OA. However, although targeting ER stress has been proven to alleviate OA progression *in vitro* and *in vivo*, the treatments for OA remain in preclinical stage and require further investigation.

Osteoarthritis (OA), the most common degenerative joint disease worldwide, is characterized by cartilage degradation, pain, and limited movement, leading to a significant reduction in the quality of life of patients. For many years, OA has been identified as a degenerative disease of cartilage; however, there is increasing evidence that OA is a whole-joint disease involving cartilage degradation, synovitis, meniscal lesions, and subchondral bone remodeling [1]. Research on the pathogenesis and treatment of OA is rapidly developing, but progress is still limited.

ER stress, the accumulation of misfolded/unfolded proteins in the ER due to an impairment in the ER’s protein-folding ability, has a detrimental effect on the physiological function and survival of most cell types [2]. Once ER stress occurs, intracellular adaptive responses (including the unfolded protein response (UPR) and ER-associated degradation (ERAD) pathways) are activated protein quality (details are available in published reviews) [3]. However, excessive and prolonged ER stress beyond the scope of the UPR and ERAD pathways can lead to cellular dysfunction and even death [3]. Recently, the role of ER stress in the pathogenesis and treatment of OA has attracted considerable attention (Fig. 1A).

## ER stress in the pathogenesis of OA

### ER stress in cartilage degradation

Excessive and prolonged ER stress can lead to chondrocyte apoptosis, while mild ER stress can activate autophagy via the GRP78 pathway and protect chondrocytes from apoptosis [4]. However, the complicated crosstalk among ER stress, autophagy, and apoptosis has not been completely elucidated. Recently, Yang et al. reported that the IRE1-mTORC1 (the mechanistic target of rapamycin complex 1) -PERK signaling pathway coordinated autophagy and apoptosis in chondrocytes of osteoarthritic temporomandibular joints (Fig. 2) [5]. CHOP is a critical switch of the UPR in regulating apoptosis, and AMPK can inhibit CHOP-mediated apoptosis [6]. Under physiological conditions, SIRT1 deacetylates PERK and attenuates the PERK-eIF-2α-CHOP axis, thereby maintaining cartilage homeostasis [7].

**Figure 1.**
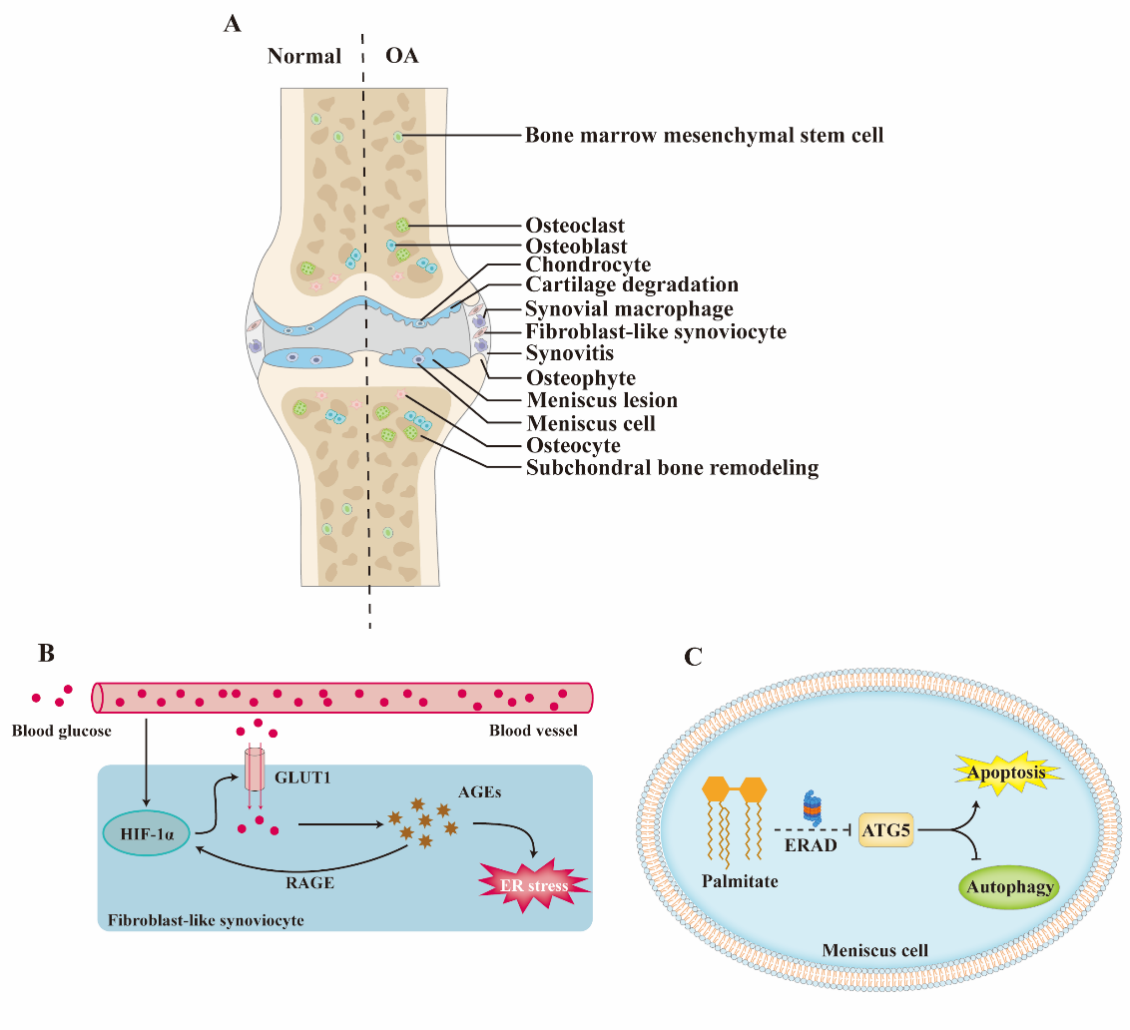
ER stress in the pathogenesis of OA. (A) The cell types involved in OA progression and affected by ER stress. (B) Mechanisms through which ER stress accelerates OA progression in synovitis. (C) Mechanisms through which ER stress accelerates OA progression in meniscal lesions.

### ER stress in synovitis

The synovium can be divided into lining layers that contain numerous fibroblast-like synoviocytes (FLSs), synovial macrophages, and the sub-lining layer, which contains more connective tissue and fewer cellular components. Li et al. found that high glucose stimulation promoted ER stress and pro-inflammatory mediator release through the HIF-1α (hypoxia-inducible factor 1α) -GLUT1 (glucose transporter 1) -AGE pathway in rat FLSs [8]. Interestingly, the binding of AGEs to the receptor for AGEs (RAGE) can activate HIF-1α signaling in multiple tissues [9]. Therefore, a vicious cycle of HIF-1α-GLUT1-AGEs-HIF-1α may exist in the FLSs of diabetes-related OA (Fig. 1B).

### ER stress in meniscal lesions

The meniscus is an important component of the joint and plays a critical role in inhibiting cartilage degradation and OA progression by reversing abnormal mechanical forces. Mallik et al. showed that palmitate degraded ATG5 through the ERAD pathway to inhibit autophagy and promote meniscus cell apoptosis (Fig. 1C) [10].

### ER stress in subchondral bone remodeling

Osteoblasts, osteoclasts, osteocytes, and bone marrow mesenchymal stem cells (BMSCs) are generally considered key cells involved in subchondral bone remodeling. Wang et al. found that OA subchondral bone remodeling was usually accompanied by decreased subchondral bone osteoblast mineralization and that the inhibition of phosphoinositide-specific phospholipase C-γ1 (PLC-γ1) can promote osteoblastic mineralization, partly due to ER stress inhibition [11]. Moreover, ER stress can regulate bone homeostasis by modulating the differentiation and function of osteoclasts and the survival of osteocytes [12, 13]. Excessive ER stress and autophagy dysfunction in BMSCs can promote inflammation-mediated bone loss [14].

**Figure 2.**
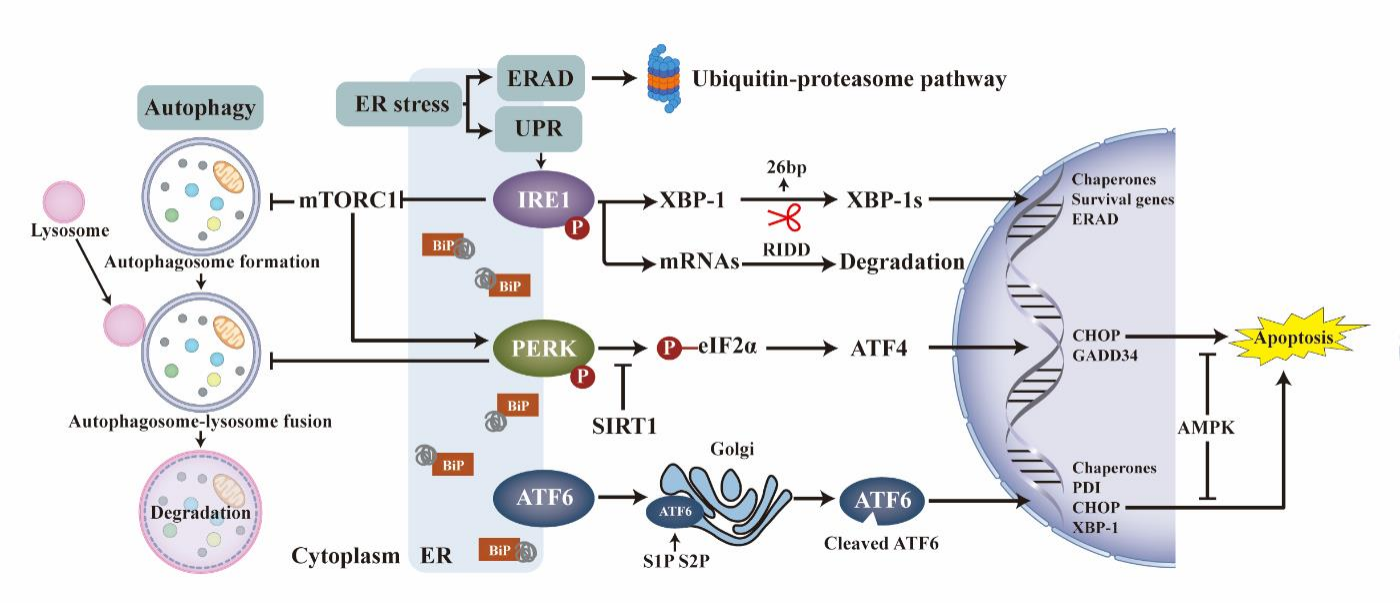
Detailed mechanisms through which ER stress accelerates OA progression in cartilage degradation.

## Treatment targeting ER stress to alleviate OA

The use of chemical chaperones to assist in protein folding is one approach for attenuating ER stress. 4-phenylbutiric acid (4-PBA), a small molecular chemical chaperone, reportedly decreases excessive ER stress, apoptosis, cytokine secretion, and cartilage damage in anterior cruciate ligament transection (ACLT) rat models [15]. Tauroursodeoxycholic acid (TUDCA), another chemical chaperone, was proven to reduce the levels of ER stress markers, recover cell proliferation, decrease apoptosis, and increase the expression of type II collagen [16]. Another strategy for targeting ER stress is the blockade of protein synthesis. Salubrinal, a selective inhibitor of eIF2α phosphatase, was found to inhibit ER stress-mediated up-regulation of MMP13 in tunicamycin-treated human chondrocytes [17].

## Perspective

Increasing evidence suggests that OA is a whole-joint disease and that its pathological changes include cartilage degradation, synovitis, meniscal lesions, and subchondral bone remodeling. ER stress influences the physiological function and survival of chondrocytes, FLSs, synovial macrophages, meniscus cells, osteoblasts, osteoclasts, osteocytes, and BMSCs through complicated signaling pathways and mechanisms involved in the abovementioned pathological changes. In addition, researchers should pay attention to ER stress in OA pain and in ligament and skeletal muscle degeneration. Targeting ER stress is expected to lay the foundation for the development of disease-modifying drugs for OA. However, the efficacy of targeting ER stress in humans is indeterminate. Further studies are warranted to explore the role and therapeutic potential of ER stress in patients with OA.
